# Weight loss induced bone loss: mechanism of action and clinical implications

**DOI:** 10.1038/s41413-025-00483-4

**Published:** 2025-12-02

**Authors:** Hanghang Liu, Bolun Li, Linyi Liu, Wangyang Ying, Clifford J. Rosen

**Affiliations:** 1 State Key Laboratory of Oral Diseases & National Center for Stomatology & National Clinical Research Center for Oral Diseases, West China Hospital of Stomatology, Sichuan University, Chengdu, Sichuan China; 2 Maine Medical Center Research Institute, Maine Medical Center, Scarborough, ME USA; 3 Department of Oral and Maxillofacial Surgery, The Affiliated Stomatological Hospital of Southwest Medical University, Luzhou, China; 4 Machine Intelligence Laboratory, College of Computer Science, Sichuan University, No. 24 South Section 1, Yihuan Road, Chengdu, China

**Keywords:** Bone, Obesity

## Abstract

Weight loss, whether resulting from disease-related conditions or intentional interventions, has been increasingly recognized as a significant risk factor for compromised skeletal integrity. While moderate weight reduction may yield metabolic benefits, rapid or sustained weight loss is frequently associated with decreased bone mineral density, deterioration of bone microarchitecture, and heightened fracture risk. The mechanisms underlying weight loss–induced bone loss are complex and multifactorial. Emerging evidence highlights a range of contributing factors, including reduced mechanical loading, increased bone marrow adiposity, hormonal and endocrine alterations, nutritional deficiencies, and disruptions in energy metabolism. These mechanisms are intricately interconnected, ultimately impairing bone remodeling and homeostatic balance. In this review, we provide a comprehensive analysis of the current literature on the mechanistic pathways, clinical consequences, and therapeutic strategies related to weight loss–induced bone loss. We further differentiate the skeletal effects of disease-associated versus intervention-induced weight loss, with a focus on their distinct molecular underpinnings. Our goal is to offer novel insights into the optimization of bone health management in the context of weight loss, guided by a translational medicine perspective.

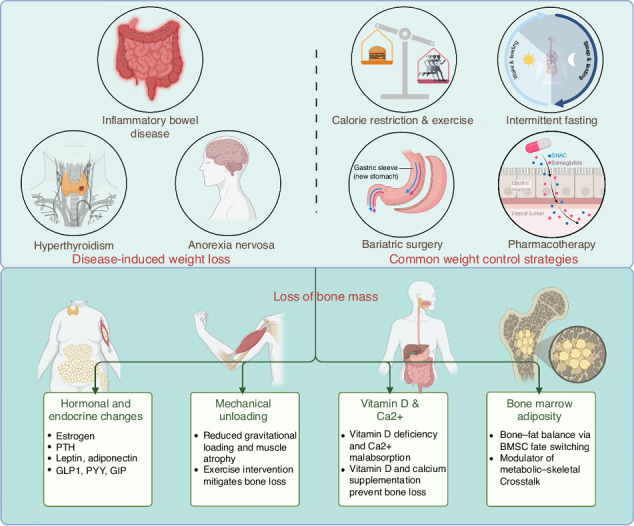

## Introduction

Weight loss can arise in a variety of clinical contexts, including chronic diseases such as anorexia nervosa and inflammatory bowel disease, as well as from active medical interventions like bariatric surgery and pharmacotherapy. While moderate weight reduction is often therapeutically beneficial—particularly in improving metabolic disorders such as type 2 diabetes and obesity—substantial or prolonged weight loss has been increasingly implicated in adverse skeletal outcomes. These encompass decreased bone mineral density (BMD), compromised bone microstructure, and an increased risk of fractures.^1^ In recent years, a growing body of research has elucidated the multifactorial mechanisms underlying weight loss–induced bone loss. Reduced mechanical loading diminishes skeletal stimulation and promotes osteoclastic bone resorption.^2–4^ Concurrently, increased bone marrow adiposity has been shown to inhibit osteoblast differentiation and function.^5,6^ Hormonal and endocrine alterations—such as reduced levels of insulin-like growth factor 1 (IGF-1), estrogen, and parathyroid hormone—disrupt the tightly regulated balance of bone remodeling.^7^ Nutritional deficiencies, commonly observed in states of malnutrition or restrictive intake, deprive the skeleton of essential substrates for bone formation and maintenance.^8^ Moreover, changes in systemic energy metabolism may impair the energetic support necessary for skeletal homeostasis and influence bone-active hormonal pathways.^9^ In this review, we systematically examine current evidence on the multifaceted mechanisms, clinical consequences, and potential intervention strategies related to weight loss–induced bone loss.

## Disease-Induced Weight Loss and Its Impact on Skeletal Health

Anorexia Nervosa (AN) is a critical disorder of eating behavior, defined by extreme calorie limitation, a persistent dread of gaining weight, and a misperception of body shape, ultimately causing markedly reduced body mass.^10–12^ Multiple studies have found that the BMD of AN patients in the active phase is lower than that of healthy controls, with a decrease of 16% in the lumbar spine, 18% in the femoral neck, and 23% in the total hip.^13^ The latest meta-analysis shows that AN adult patients have an average reduction of 0.16 kg in whole-body bone mineral content,^14^ and there is no gender difference in the reduction of BMD in adolescent patients,^15^ In terms of bone microstructure, abnormalities are observed in the distal radius and distal tibia of AN patients, manifested as reduced volumetric bone mineral density (vBMD), decreased cortical bone area and thickness, increased porosity, decreased trabecular bone density and increased separation degree, but the results of studies on trabecular bone number changes are inconsistent.^16–18^

Fracture risk is high among AN patients. In one study of 208 patients, 45 experienced 88 fractures, with Standardized Incidence Ratios (SIRs) of 2.9 in females and 3.4 in males.^19^ Another study (*n* = 803) reported an incidence rate ratio (IRR) of 2.2, with elevated fracture risks at the vertebrae (3.8), upper arm (3.0), and hip (6.6).^20^ A large UK cohort (9 239 females, 556 males) found higher fracture risk in females across all ages (HR = 1.59) and in males over 40 (HR = 2.54), with hip/femur and pelvis most affected in females, vertebrae in males.^12^ Mechanistically, AN increases bone marrow adiposity and impairs osteogenesis, with fat fraction in femoral neck and vertebrae inversely correlated with total hip BMD.^21,22^

Weight loss from Inflammatory bowel disease (IBD), is common. IBD is a chronic relapsing-remitting inflammatory condition encompassing ulcerative colitis (UC) and Crohn’s disease (CD), affecting approximately 6.8 million patients globally in 2017.^23,24^ Long-term follow-up studies indicate that the incidence of osteoporosis in IBD patients is higher than that in the control population, with around 14.2%–20.1% of IBD patients and 6.6% of the control group being diagnosed with osteoporosis.^25–27^ A systematic review revealed that the prevalence of osteoporosis in IBD patients ranged from 2% to 15%, and CD patients may have a higher risk than UC patients.^28^ Bone microstructural changes in the tibia, fibula, and radius are common in IBD.^29–31^ CD patients exhibit more severe cortical and trabecular loss than UC patients, whose deficits are mostly limited to cortical bone.^31^ Another study also confirmed greater trabecular separation abnormalities in CD than UC.^29^ Together, these findings indicate that CD leads to more extensive and pronounced bone microstructural damage.

IBD, is also frequently associated with low bone mineral density. The pathogenesis of IBD-related bone loss is multifactorial and is related to chronic inflammation, malabsorption, changes in the gut microbiota and glucocorticoid administration. IBD alters the bone microenvironment by suppressing osteoblasts and activating osteoclasts, leading to reduced bone density and impaired microstructure.^32,33^ As IBD often begins in youth, it may hinder peak bone mass acquisition and elevate adult fracture risk.^28,34^ Fractures commonly occur in the forearm, followed by the arm, hand, and spine. In one study, 36% of 102 IBD patients reported fractures, with five vertebral fracture cases.^30,35^ A meta-analysis estimated a 32% higher osteoporotic fracture risk in IBD patients versus controls.^36^

Hyperthyroidism is a common endocrine disorder with a global prevalence of about 0.2%–2.5%.^35,37^ The disease can widely affect multiple systems, including cardiovascular, nervous, and skeletal systems. Excess thyroid hormone stimulates greater bone turnover, and is mainly manifested as increased osteoclast activation leading to increased bone resorption, decreased bone formation, reduced BMD, and increased fracture risk. The effect of excess thyroxine on osteoblast function is less clear but the imbalance between formation and resorption is evident and is associated with increased expression of RANKL and OPG^38,39^ Table 1).

**Table 1:** Disease that induced weight loss and its related impact on bone metabolism

Disease		Impact on Skeletal Health		
		BMD	Microstructure	Fracture risk
Anorexia Nervosa		Lumbar spine, femoral neck, and total hip aBMD decreased by 16%, 18%, and 23% respectively^13^; More severe bone loss in AN-R compared to AN-BP^15^	vBMD decreased by 18%, cortical thickness by 13%, porosity increased by 46%, and trabecular separation by 11% in radial. vBMD decreased by 21.5%, trabecular number by 5.3%, and trabecular spacing increased by 11.6% in tibia.^16–18^	Vertebral, upper arm, and hip fracture incidence increased by 380%, 300%, and 660% respectively.^20^ Increased fracture risk: 159% for females, 254% for males over 40.^12^
Chronic Inflammatory Bowel Disease	ulcerative colitis	aBMD decreased by 2% in radius, 7% in femoral neck, 7% in total hip, and 7% in lumbar spine^30^	Cortical and trabecular bone structure abnormalities in the tibia, fibula, and radius, and Crohn’s disease patients have more extensive and severe bone damage^29–31^	Fracture risk increased by 32%^36^; with Crohn’s disease showing a greater incidence than ulcerative colitis ( > 10% increase)^33^
	Crohn’s disease			
Hyperthyroidism	Graves’ disease	aBMD decreased by 12.5% in lumbar spine, 14.6% in femoral neck, 16.3% in total hip, and 15.6% in radius^191^	74% bone microstructure is degenerated^191^	-
	Toxic nodular goiter	aBMD decreased by 18.85% in total spine (males), 13.33% (premenopausal females), and 21.22% in femur total (males), 7.94% (premenopausal females)^192^	-	-
	Subclinical hyperthyroidism	Some studies show reduced aBMD in the lumbar spine and hip, but others show no significant difference^193,194^	-	The risk of fracture is increased 38%^195^

## Influence of Common Weight Control Strategies on Bone Metabolism

### Calorie Restriction

Caloric restriction (CR) is a dietary regimen designed to reduce energy intake with the objective of achieving weight loss and improving metabolic health.^40^ Nevertheless, accumulating evidence indicates that CR is detrimental to skeletal metabolism, as evidenced by reductions in BMD and bone mineral content (BMC), diminished bone formation indices (e.g., bone formation rate, osteoblast number), and a concomitant increase in bone resorption.^41^

Clinical studies have demonstrated that CR-induced weight loss influences BMD in a time- and site-dependent manner. For instance, in the CALERIE trial, which involved healthy middle-aged adults undergoing a modest caloric restriction of approximately 12% over two years, areal BMD was observed to decline by 1% to 2% annually at the spine and hip.^42^ Likewise, a systematic review of 41 studies involving overweight or obese participants showed a significant reduction in total-hip BMD (0.010–0.015 g/cm^2^) after weight-loss interventions lasting six months or longer, whereas lumbar-spine BMD remained stable.^43^ Whole-body BMD declined significantly only at the six-month assessment, with no consistent changes observed at subsequent time points. Complementing these findings, a one-year randomized controlled trial by Villareal and colleagues demonstrated that obese older adults who achieved a 10.1% weight loss through combined diet and exercise experienced significant decreases in hip BMD—including the trochanter and femoral neck—while spine and whole-body BMD were preserved.^44^ This site-specific sensitivity to weight loss may be attributed to inherent differences in bone composition and response to loading. The more pronounced decline in hip BMD, as compared to the relative preservation of lumbar spine BMD, is likely due to the predominance of mechanically responsive cortical bone in the hip, whereas the lumbar spine is composed largely of metabolically active trabecular bone. Furthermore, assessments of lumbar spine BMD can be affected by age-related degenerative changes, which can obscure or underestimate the extent of true bone loss in this region.^45^

Compartment-specific differences in volumetric BMD have also been observed in response to CR interventions. Beavers et al. reported that after 18 months of CR, the trabecular bone score at the hip decreased by 0.22%, while cortical thickness declined by 2.45%. Similarly, Schoell et al. found a 6.6% decrease in lumbar spine vBMD —although this change was not statistically significant—with no significant alterations in cortical thickness. These findings highlight regional heterogeneity in skeletal responsiveness to CR interventions.^2,46^

Long-term follow-up studies have indicated an elevated fracture risk associated with CR. In a median 11.3-year prospective study involving 5 145 individuals with type 2 diabetes, Lewis et al. reported that participants in the CR combined with exercise group experienced significantly higher rates of hip fractures (10 per 10 000 person-years) compared to controls (6 per 10 000 person-years). The incidence of fragility fractures—including those of the hip, pelvis, or upper arm/shoulder—was also increased in the intervention group (35 vs. 25 per 10 000 person-years), corresponding to a 39% higher relative risk.^47^

During the initial phase of CR interventions, serum markers of bone turnover—such as osteocalcin, C-terminal telopeptide of type I collagen (CTX), and N-terminal telopeptide (NTX)—increase significantly, reflecting an acceleration of bone resorption.^43^ A two-year randomized controlled trial by Villareal et al. demonstrated that, relative to the ad libitum group, the CR group showed markedly higher levels of bone resorption markers (CTX and TRAP5b), accompanied by a notable decrease in the bone formation marker bone-specific alkaline phosphatase (BAP). These findings suggest that CR may disrupt the balance between bone formation and resorption, favoring a catabolic skeletal state.^42^ Interestingly, findings from an ultra-long-term CR study (mean duration: 6.8 years; ~35% CR) revealed unexpected outcomes. Although the CR group exhibited significantly lower lumbar spine and hip BMD compared to controls, there were no significant differences in serum CTX, bone-specific alkaline phosphatase (BSAP) levels, or trabecular microarchitecture parameters (e.g., erosion index, surface-to-volume ratio) between the groups.^48^ These results suggest that prolonged CR may influence bone metabolism through mechanisms distinct from those observed in short-term interventions, highlighting the need for further investigation. On the other hand, it might also reflect a new steady state in which bone turnover is suppressed with long-term energy depletion, not unlike the skeletal changes in mice with CR.

Animal studies reinforce compartment-specific skeletal changes with CR. In young male mice (3 weeks of age), nine weeks of CR significantly suppressed both trabecular and cortical bone accrual in the femur.^49^ In contrast, female mice aged 6 weeks or older exhibited marked reductions in cortical, but not trabecular, bone following CR.^50^ Consistent with these sex and compartment-dependent effects, our preliminary data showed that an 8-week, 30% CR regimen induced cortical bone loss in both sexes, whereas trabecular bone mass was enhanced in females.^51^ Similarly, Behrendt et al. examined long- and short-term CR in femora and vertebrae, demonstrating that the initially deleterious impact of CR on cancellous bone diminishes with age. Notably, in the lumbar spine, prolonged CR (20 or 74 weeks) yielded graded benefits in trabecular volumetric BMD, bone volume fraction, and trabecular number.^52^

Although the mechanisms underlying the sex- and compartment-specific skeletal effects of CR remain incompletely defined, several plausible explanations can be advanced. Regarding sex differences, sex hormones are essential for skeletal homeostasis, and CR may disrupt estrogen signaling more profoundly in females, thereby adversely affecting bone metabolism. CR also promotes expansion of bone-marrow adipose tissue (BMAT), which suppresses osteoblastogenesis; this effect appears to be more pronounced in females owing to sex-specific regulation of adipogenesis. Differences in skeletal maturation may further contribute, as females reach skeletal maturity earlier than males, rendering the timing of CR relative to growth phases critical for site-specific vulnerability. Moreover, the greater lean mass and mechanical loading typically observed in males may provide partial protection against CR-induced bone resorption.

Compartment-specific skeletal responses to CR likely arise from fundamental differences between trabecular and cortical bone. Owing to its high surface area and metabolic activity, trabecular bone is highly sensitive to rapid hormonal and nutritional fluctuations, making it initially more susceptible to CR-induced remodeling alterations. However, prolonged CR may diminish turnover in this compartment, occasionally yielding modest gains in trabecular indices as seen in female C57BL6J mice undergoing 30% CR during their vulverable period of accelerated bone resorption (i.e., between 8 and 16 weeks). By contrast, cortical bone—which is responsible for the majority of load-bearing capacity—appears particularly vulnerable to CR-induced increases in porosity. Energy deficiency may enhance intracortical remodeling and reduce periosteal bone apposition, leading to a net loss of cortical thickness and strength. In addition, CR (or aging) can promote ‘trabecularization’ of the endocortical surface, whereby cortical bone acquires trabecular-like characteristics, further compromising mechanical competence. Differential vascularization, marrow composition, and osteocyte density between trabecular and cortical compartments likely modulate these divergent responses, collectively explaining the distinct patterns of bone loss observed under CR in mice. Studies of CR focused on osteocyte responsiveness are lacking but may be essential to understand the cortical bone changes that are evident in mice and humans (Table 2).

**Table 2:** Common weight control strategies and related impact on bone metabolism

Weight Control Strategies		Impact on Bone Metabolism			
		BMD	Microstructure	Fracture risk	BTMs
Calorie Restriction		Decreased hip aBMD by 0.010 to 0.015 g/cm^2^, with no significant change in lumbar spine aBMD^43,44^	Decreased vBMD in the hip by 9.09%, with a non-significant decline in lumbar spine vBMD^2,46^	Increased rate of fragility fractures (hip, pelvis, or upper arm/shoulder fractures) and elevated fracture risk by 39%^47^	Bone resorption markers CTX increased by up to 28.49% and TRAP5b by up to 28.57%, while bone formation marker BASP decreased by up to 6.91%^42^
Intermittent Fasting	Time-restricted eating	No notable influence^62^	-	-	No notable influence^63^
	Alternate-day fasting	Some studies show reduced lumbar spine aBMD ^61^,^62^, while others show no significant effect^58,59^	-	-	No notable influence^59^
Semaglutide Treatment		Reduced lumbar spine aBMD by 2.05% and total hip aBMD by 2.59% (at non-weight-loss doses)^72^ In ovariectomized osteoporosis rat models, spine BMD rose by 15.65%–29.3%, femur BMD by 42.39%–48.7%, and tibia BMD by 23.35%–25.6%.^73^ In HFD-induced obese mice, BMD of the whole body and various parts was unaffected.^75^	Tibial vBMD decreased by 1.69%, and cortical thickness reduced by 1.75%^72^ In type 2 diabetic mice, femoral cortical thickness and maximum load decreased^74^	No notable influence^67–69^	Significant increase in bone resorption marker CTX by 54.8% (at non-weight-loss doses)^72^ In type 2 diabetic mice, RANKL mRNA expression decreased while OPG mRNA expression increased.^74^ In ovariectomized osteoporosis rat models, Semaglutide cut CTX by 46.1%–54.76%, PYD by 39.1%–59.4%, BALP by 55.6%–64.96%, and OCN by 67.48%.^73^ In HFD-induced obese mice, BMD of the whole body and various parts was unaffected.^75^
Vertical Sleeve Gastrectomy		Significant decrease in femoral neck BMD by 1.5%–3.32% and total hip aBMD by 1.5%–9.2% ^80,81,84^, while lumbar spine aBMD showed either an increase^80,85^ or inconsistent changes across studies^81,82,84^	vBMD decline in lumbar spine, radius, and tibia by 0.4%–3.1%, trabecular bone deterioration by 3.3%–10%, and elevated cortical porosity by 0.3%–5.5% with decreased cortical thickness by 0.1%–1.6% in tibia and radius^82^	Fracture incidence reduced by 11.92%, but fracture risk was not statistically significant^87^	Bone resorption and formation markers increased significantly by 27%–200%^94^,^95^, but bone formation and resorption remained relatively balanced^89^
Exercise	Aerobic exercise	Whole-body aBMD increased by 0.96%–2%, pelvic aBMD by 1.85%–2.63%^99,101^	-	-	Serum osteocalcin levels increased by 11.92%–45.45%^100^
	Resistance exercise	Whole-body and lumbar spine BMD increased^101,103^ or had no significant effect^102^	-	-	-
	Combined exercise	No significant effect on whole-body, lumbar spine, and hip BMD^104,105^	-	-	-

### Intermittent Fasting

Intermittent fasting (IF), a dietary strategy characterized by periodic restriction of energy intake, enhances fat oxidation and metabolic efficiency through metabolic switching, offering significant benefits for weight management, insulin sensitivity, and lipid profiles.^53^ Unlike traditional continuous caloric restriction, IF involves alternating cycles of marked energy limitation with ad libitum feeding periods.^54^ The principal IF regimens include: (1) the 5:2 diet (75% energy restriction on two non-consecutive days per week), (2) alternate-day fasting (ADF), (3) modified ADF, and (4) time-restricted eating (TRE). Among these, TRE—limiting the daily eating window to 4–12 h, typically with ≥12-h overnight fasting—has gained popularity due to its practical feasibility and better adherence. While ADF is effective for weight reduction, it is associated with lean mass loss and poor long-term compliance.^55^

Evidence regarding the impact of IF on bone health remains inconsistent. In terms of ADF, cross-sectional studies suggest that skipping breakfast may be associated with reduced bone density; for example, a Japanese cohort study showed that women who skipped breakfast ≥3 times a week had significantly lower hip bone density,^56^ and young men exhibited increased risk of lumbar bone loss.^56,57^ However, a 6-month ADF trial by Stekovic et al. found no significant changes in whole-body or lumbar bone density.^58^ Additionally, a 6-month ADF intervention by Barnosky et al. reported no significant effects on BMC, BMD or bone turnover markers (such as osteocalcin, CTX, IGF-1).^59^ Regarding TRE, TRE trials generally report no impact on BMC after a 3-month intervention.^60,61^ Similarly, a 6-week TRE intervention in middle-aged adults showed no changes in BMD.^62^ However, Lobene et al. observed that in the TRE group, the decline in the bone formation marker P1NP was significantly attenuated (-0.22 vs -4.07 ng/mL), indicating potential bone-protective effects.^63^

Few in vivo studies have been conducted to investigate the potential effect of IF on bone metabolism. In a glucocorticoid-induced osteoporosis (GIO) rat model, IF (16–18 h of fasting per day for 90 consecutive days) demonstrated significant protective effects against bone loss.^64^ A 90-day administration of dexamethasone (DEX) resulted in significant declines in both BMD and BMC, along with substantial increases in serum levels of glucose, insulin, triglycerides, total cholesterol, parathyroid hormone (PTH), osteoprotegerin (OPG), and bone resorption markers including deoxypyridinoline (DPD), N-terminal telopeptide of type I collagen (NTX-1), and TRAP-5b. Additionally, serum levels of bone formation markers—ALP and OCN—as well as RANK were significantly suppressed. IF intervention effectively reversed these alterations, restoring BMD and BMC while reducing bone resorption markers and enhancing bone formation indices. These results suggest that IF ameliorates glucocorticoid-induced bone loss primarily by suppressing osteoclastogenesis and PTH secretion and promoting osteoblastic activity. Moreover, another study investigated the skeletal effects of IF in the context of a ketogenic dietary regimen.^65^ While the traditional ketogenic diet (KD) has long been used for obesity management, it has also been associated with adverse effects on bone health, including compromised bone mass and structural integrity. To evaluate whether integrating IF into a ketogenic framework might mitigate these skeletal effects, rats were fed either a continuous KD or an every-other-day ketogenic diet (EODKD)—a regimen combining KD with IF. After 12 weeks, both KD and EODKD groups exhibited elevated ketone bodies, increased body fat percentage, and reduced body weight relative to controls. Importantly, both dietary interventions negatively affected bone mass and mechanical strength. Compared to continuous KD, every-other-day ketogenic diet (EODKD) induced a greater degree of ketosis but was associated with a tendency to suppress osteoclast activity and hinder early osteogenic differentiation of BMSCs, as indicated by TRAP and ALP staining, along with alizarin red assays. These results imply that while EODKD does not completely avert the bone deficits linked to KD, it may differentially modulate bone remodeling—dampening bone resorption while mildly impairing early osteoblast development.

### Pharmacotherapy: semaglutide treatment

GLP-1 Receptor Agonists (GLP-1 RAs) are a group of medications used to treat type 2 diabetes and obesity by mimicking the incretin hormone GLP-1, thereby promoting blood glucose reduction and weight loss, along with providing added advantages such as lowering cardiovascular and renal risk. These medications are categorized into short-acting and long-acting formulations (e.g., liraglutide, exenatide, dulaglutide, albiglutide, semaglutide).^66^ Notably, semaglutide and tirzepatide (GLP-1RA + GIPR agonist) have been approved for chronic weight management in non-diabetic obese patients due to its significant weight-reducing efficacy.^45^ These agents are also approved for the treatment of T2D.

The impact of GLP-1 RAs on bone health has been explored in multiple clinical studies, though findings are inconsistent. A meta-analysis conducted by Mabilleau et al. revealed that GLP-1RA therapy did not reduce fracture incidence in type 2 diabetic patients.^67^ A Bayesian network meta-analysis pooled data from 54 RCTs involving 49 602 participants, of whom 28 353 received GLP-1 RA treatment demonstrated that GLP-1 RAs were associated with a reduced risk of fractures compared to placebo or other antihyperglycemic agents. Among the agents assessed, exenatide demonstrated the greatest risk reduction (RR = 0.17; 95% CI: 0.03–0.67), followed in order by dulaglutide (1.04%), liraglutide (1.39%), albiglutide (5.61%), lixisenatide (8.07%), and semaglutide (18.72%).^68^ However, exenatide is rarely still used because it is associated with very minimal weight loss. A separate meta-analysis including 117 RCTs with 221 364 participants revealed that albiglutide (RR = 0.29; 95% CI: 0.04–0.93) and voglibose (RR = 0.03; 95% CI: 0–0.11) were associated with significantly lower fracture risk, whereas trelagliptin (a DPP-4 inhibitor) was linked to increased fracture risk (RR = 3.51; 95% CI: 1.58–13.70). Other GLP-1 RAs, showed neutral effects.^69^ Another meta-analysis of RCTs revealed that GLP-1 receptor agonist therapy lasting more than 52 weeks was linked to a significantly lower risk of fractures compared to placebo or alternative antidiabetic treatments.^70^

However, trials examining bone turnover markers and BMD provide a more nuanced view. In a 52-week randomized, double-blind, placebo-controlled phase 2 trial involving 64 adults at high fracture risk (due to low BMD or recent fragility fractures), participants received once-weekly semaglutide (1.0 mg) or placebo. Although no significant change was observed in the bone formation marker P1NP, the semaglutide group showed a significant increase in the bone resorption marker CTX and a reduction in areal BMD at both the lumbar spine and total hip. In a separate 12-month prospective study of 65 patients with type 2 diabetes mellitus (T2DM) initiating GLP-1 RA therapy (dulaglutide or semaglutide), significant reductions in body weight and BMI were also observed.^71^ Bone turnover markers and adiponectin levels increased, while myostatin decreased modestly. Femoral BMD decreased modestly but significantly, indicating that GLP-1 RAs may activate bone resorption and potentially reduce bone mass, particularly with concurrent weight loss.

Use of GLP-1 Ras, in people with obesity (PwO) may also have skeletal impacts in part because obesity may itself predispose to altered micro-architecture and greater fracture risk. A systematic review and meta-analysis of 12 randomized controlled trials (RCTs) involving 11 459 overweight or obese adults without diabetes demonstrated that GLP-1 receptor agonists significantly reduced body weight, alongside notable improvements in glycemic control, lipid levels, and blood pressure. In one study, liraglutide treatment was associated with a decrease in both fat mass and lean mass, as well as a small but statistically significant reduction in whole-body BMD (mean difference = −0.02 kg/m^2^; *P* = 0.03). In a recent 1 year trial by Hansen et al. semaglutide drove a significant reduction in femoral BMD that was associated with lower cortical thickness, but no changes in trabecular bone by extreme CT.^72^

Animal studies offer some mechanistic insights into the skeletal effects of GLP-1 RAs. In an OVX rat model of postmenopausal osteoporosis, subcutaneous administration of semaglutide (150 or 300 μg/kg for 10 weeks) significantly improved trabecular bone microarchitecture and preserved bone mineral content.^73^ The treatment increased β-catenin expression and suppressed RANKL activation, suggesting that semaglutide may promote osteoblastogenesis and inhibit osteoclast-mediated bone resorption through the Wnt signaling pathway. In a study using a T2DM mouse model induced by streptozotocin and high-fat diet, semaglutide and tirzepatide both reduced blood glucose levels. However, cortical bone thickness was decreased in the semaglutide group (*P* = 0.032), while no significant changes in bone microarchitecture or biomechanical strength were noted. Although bone turnover markers CTX and P1NP declined, differences among groups were not statistically significant.^74^ In a comparative study in obese male mice of vertical sleeve gastrectomy (VSG), semaglutide treatment, and controls, both interventions improved glycemic control and reduced body weight, but VSG significantly decreased energy expenditure and induced notable bone loss, whereas semaglutide preserved BMD over six weeks.^75^

### Bariatric surgery: VSG

Bariatric surgery is used to treat obesity and its related comorbidities. The types include VSG, Roux-en-Y gastric bypass (RYGB), laparoscopic adjustable gastric banding (AGB), intragastric balloon placement, gastric plication and suturing, and duodenal-jejunal bypass liner implantation, among others.^76^ VSG involves resection of the majority of the stomach to create a slender tubular gastric pouch, thereby reducing gastric capacity and limiting food intake to achieve weight loss.^77^ Due to its procedural simplicity, significant weight loss efficacy, and minimal impact on intestinal absorption, VSG has demonstrated an upward trend in clinical application in recent years, emerging as the most prevalent bariatric surgical procedure.^76^

Numerous studies have investigated the effects of bariatric surgery on BMD, though findings vary across studies. A 2015 meta-analysis by Ko et al.^78^ revealed that patients who underwent bariatric surgery had significantly lower femoral neck BMD compared to non-surgical controls, whereas lumbar spine BMD did not differ significantly between the groups. Additionally, Carmona et al.^79^ conducted another systematic review with categorical analysis, finding that patients in the malabsorptive surgery group (including RYGB and biliopancreatic diversion) experienced significant BMD reduction one year postoperatively, particularly at the hip and spine. In contrast, the restrictive surgery group (including VSG and gastric banding) showed smaller BMD changes, with even a slight increase in total BMD. This discrepancy may stem from malabsorptive procedures altering digestive pathways and causing nutrient malabsorption, whereas restrictive surgeries primarily limit food intake through gastric volume reduction with minimal impact on digestion and absorption.

#### Changes in BMD

Studies on BMD changes following VSG demonstrate site-specific and population-dependent variations. Multiple studies indicate a general decline in hip BMD post-VSG, though the extent varies. Studies^80–83^ reported a 1.7%–3.32% and 1.5%–8% reduction in femoral neck and total hip BMD, respectively, in male patients at 6–12 months postoperatively, attributing these changes to decreased mechanical loading from weight loss. It was also noted that postmenopausal women exhibited more pronounced declines than premenopausal women or men.^82^ Moreover, Maghrabi et al.^84^ documented even more substantial reductions, with total hip BMD decreasing by 7.6% and 9.2% at one and two years postoperatively, indicating persistent bone loss. By contrast, lumbar spine and radial BMD changes are less consistent and usually remined stable.^83^ suggesting mechanical unloading as a primary contributor. Adamczyk et al.^80^ and Tovar et al.^85^ observed significant lumbar BMD increases (2.89% at six months, 5.7% at one year, 7.9% at two years), potentially linked to improved vitamin D levels and reduced parathyroid hormone (PTH). Conversely, other studies^81,82^ reported a 1.4%–2.1% decrease in lumbar aBMD at 12 months, and Maghrabi et al.^84^ noted minor declines (0.7% at one year, 2.3% at two years), though unmeasured vitamin D and PTH levels complicate interpretation.

#### Bone microstructure

High-resolution peripheral quantitative computed tomography (HR-pQCT) revealed declines in vBMD at both the radius and tibia, with particularly pronounced reductions in total and cortical vBMD at the tibia in postmenopausal women.^82^ Microstructural analyses showed trabecular thinning, increased separation and heterogeneity—changes most marked in postmenopausal subjects. Cortical bone demonstrated elevated porosity and reduced tibial cortical thickness, resulting in declines of 5.7%, 5.5%, and 5.2% in tibial compressive strength, stiffness, and apparent modulus, respectively.^82^ In contrast, adolescents exhibited distinct skeletal adaptations: significant reductions in tibial cortical area, thickness, and trabecular number by 12 months, accompanied by increased trabecular separation. At the radius, trabecular vBMD declined, whereas cortical vBMD increased, potentially reflecting reduced cortical porosity; however, bone strength estimates remained stable.^83^ Furthermore, Bredella et al. reported post-VSG increases in lumbar BMAT alongside peripheral MAT reductions, underscoring region-specific fat–bone interactions, although other studies report a reduction in BMAT with VSG.^86^

#### Fracture risk

A large French cohort study involving 14 253 VSG patients reported an overall major osteoporotic fracture incidence of 1.70 per 1 000 person-years, with specific rates for distal forearm/wrist (0.82), proximal humerus (0.37), clinical vertebral (0.29), and hip fractures (0.22).^87^ Although this rate was slightly lower than that observed in non-surgical controls (1.93 per 1 000 person-years), Cox regression analysis indicated no statistically significant difference in fracture risk (HR = 0.96, 95% CI: 0.83–1.11), suggesting that VSG may not substantially increase fracture incidence.^87^ In contrast, a meta-analysis by Holanda et al.^88^ found that while VSG was associated with lower fracture risk than malabsorptive procedures such as RYGB, it still conferred a modestly elevated risk compared to non-surgical controls (OR = 1.23, 95% CI: 1.05–1.44). These findings underscore the need for more longer-term, higher-quality studies.

#### Bone turnover marker dynamics

VSG induces a consistent activation of bone turnover across age groups, characterized by elevations in both resorption and formation markers.^89,90^ In adults, CTX increases by 106%–156%, PINP by 58%–59%, TRAcP5b by 27%, and total/uncarboxylated osteocalcin by 60% and 121%, respectively, within six months postoperatively.^82,89^ Notably, the PINP/CTX and CTX/TRAcP5b ratios remain near baseline at six months, suggesting a relative balance between bone formation and resorption during this period.^89^ This hypermetabolic bone state peaks at 12 months (CTX + 200%) and remains elevated above preoperative levels through 24 months.^82,90–92^ Adolescents exhibit a similar trajectory, with significant increases in osteocalcin and CTX observed between 6 and 12 months following VSG, although CTX tends to plateau after six months.^93^ In contrast, markers of mineral metabolism—including 25-hydroxyvitamin D, bone-specific alkaline phosphatase (BALP), and PTH—remain largely stable over the long term,^94^ suggesting that VSG-mediated skeletal remodeling occurs independently of traditional calcium–phosphorus regulatory pathways.

### Exercise

The impact of exercise on weight loss, particularly on a low-calorie diet can be impressive. On the other hand, changes in bone are more nuanced. Multiple studies have shown that exercise interventions can significantly enhance BMD at critical skeletal sites—including the lumbar spine, femur, and hip—thereby strengthening bone mechanical properties and lowering the risk of fractures associated with osteoporosis.^95–97^ However, existing research has primarily focused on osteoporotic models, with relatively limited researches studying the impact of exercise interventions on bone parameters in obese populations, particularly those undergoing simultaneous dietary restriction.^98^ Aerobic exercise has been shown in multiple studies to increase BMD in obese individuals. One study reported that 12 weeks of endurance training significantly improved lower limb and whole-body bone mineral content, as well as whole-body and pelvic BMD in obese participants.^99^ Another 8-week aerobic exercise intervention demonstrated an 11.92% and 45.45% increase in serum total osteocalcin and undercarboxylated osteocalcin levels, respectively, in the exercise group, and no dramatic changes were observed in the control group.^100^ Berro et al. found through a 12-month endurance exercise intervention that obese participants showed improvements in lumbar spine and whole-body BMD, along with a slight enhancement in TBS.^101^

The impact of resistance exercise on bone metabolism in obese individuals remains a subject of debate. However, a randomized controlled trial by Berro et al. demonstrated that 12 months of resistance training led to significant increases in lumbar spine and whole-body BMD among obese participants, along with improvements in TBS and enhanced femoral neck bone strength, as evidenced by gains in strength index, bending strength index, compression strength index, and impact strength index.^101^ However, Cunha et al. reported that 12 weeks of resistance training at different intensities had no significant effect on BMD in elderly women with osteoporotic obesity.^102^ In contrast, Huang et al.‘s RCT found that 12 weeks of resistance training increased BMD by an average of 5.94% in elderly women with sarcopenic obesity.^103^ These discrepancies may stem from differences in participants’ baseline conditions (osteoporosis vs. sarcopenia), resistance training protocols (type, intensity, and duration), diet, and sample size limitations. Notably, two studies on combined aerobic and resistance exercise interventions showed no significant effects on bone metabolism. Choquette et al.‘s 6-month intervention (3 sessions/week of combined resistance and aerobic exercise) in overweight/obese postmenopausal women found no improvements in whole-body, lumbar spine, or hip BMD (including total hip, femoral neck, trochanter, and Ward’s triangle).^104^ Similarly, Bolam et al.‘s 9-month combined exercise intervention (upper-body resistance + varying doses of impact loading) in middle-aged and elderly obese men revealed minimal changes ( ± 1%) in whole-body, lumbar spine and femoral neck BMD, with no between-group differences in bone turnover markers (BAP and CTX-1).^105^

Beavers et al. conducted a randomized controlled trial in obese older adults with cardiovascular or metabolic conditions, comparing three intervention groups: caloric restriction alone (WL), caloric restriction combined with aerobic training (WL + AT), and caloric restriction combined with resistance training (WL + RT).^2^ After 18 months, the WL, WL + AT, and WL + RT groups achieved weight reductions of 5.7 kg, 9.9 kg, and 10.1 kg, respectively. Although no significant differences were found among the groups in total hip and femoral neck aBMD or trabecular bone score (TBS), changes in lumbar spine aBMD approached statistical significance. Specifically, the WL and WL + RT groups showed increases of 0.012 g/cm² and 0.008 g/cm², respectively, whereas the WL + AT group experienced a decrease of 0.006 g/cm². Additionally, CT scans revealed declines in vBMD across all groups, with the smallest reduction in the resistance group, suggesting a potential protective effect against bone loss. At 30-month follow-up, despite weight regain in all groups (WL + AT: −7.1 kg; WL + RT: −6.6 kg vs. WL: −3.4 kg), the resistance group still demonstrated some bone preservation. A more recent study by Beavers et al. of older individuals undergoing dietary restriction and weighted vest or resistance training demonstrated that neither blunted the loss of bone mineral density.^106^ Villareal et al. compared control, aerobic, resistance, and combined exercise groups (aerobic + resistance), finding that after 6 months, exercise groups achieved an average 9% weight loss (8–9 kg) versus only 0.9 kg in the control group. Regarding bone metabolism, the resistance and combined groups showed significantly smaller declines in hip BMD (femoral neck, greater/lesser trochanter) compared to the aerobic and control groups, further supporting the advantages of resistance exercise in maintaining bone mass.^107^ Notably, the aerobic group exhibited significant increases in bone turnover markers (CTX, PINP, OSC), suggesting that aerobic exercise may simultaneously enhance both bone formation and resorption processes.

Animal studies provide important evidence that exercise can help mitigate bone loss associated with energy restriction or weight loss in obesity. In skeletally mature female rats, moderate energy restriction over 12 weeks had minimal negative effects on bone, but severe restriction significantly reduced bone mass and levels of IGF-1, leptin, and estradiol. When exercise was added, these negative effects were largely prevented: exercised rats had higher total bone mineral content, lean mass, and serum IGF-1, and their bone mechanical strength was maintained despite reduced energy availability.^4^ Another study in obese male rats found that while a calorie-reduced diet alone led to decreased trabecular thickness and impaired bone remodeling in the tibia and spine, adding moderate treadmill exercise improved tibial BMD and reduced bone resorption, as shown by lower CTX levels and fewer osteoclasts. However, the diet-induced bone microarchitectural deficits were not fully reversed.^108^ Overall, these studies suggest that although weight loss from dietary restriction can compromise skeletal health, incorporating exercise—particularly endurance training—can preserve BMD and hormonal factors, and reduce bone resorption, thereby offering partial protection to the skeleton during obesity treatment.

## Mechanisms Underlying Weight Loss-Induced Bone Loss

Numerous human studies have demonstrated that both disease-induced weight loss and common weight control strategies may have significant negative impacts on the skeletal system. Several potential mechanisms may be operative.

### Mechanical unloading

Bone is a mechanosensitive organ that relies heavily on continuous mechanical stimulation to maintain its structure and function. Such stimuli are derived from two primary sources: gravitational loading due to body mass and dynamic mechanical strains transmitted via muscle contractions.^3,4,109^ This loading generates ground reaction forces and strain gradients that activate mechanosensitive bone cells, particularly osteocytes, via deformation, fluid shear, and piezoelectric signaling. Weight reduction decreases vertical force transmission, leading to diminished stimulation of osteoblasts and subsequent net bone resorption. This effect is particularly evident in clinical studies of patients undergoing bariatric surgery. For instance, one study employing accelerometer-based assessments of gravitational load demonstrated that patients who lost weight following gastric bypass experienced a drop of 2 215 kN/d in cumulative daily loading within the first month, despite increased step counts. Importantly, this reduction in loading coincided with significant declines in BMD at the total hip ( − 7.0%), femoral neck ( − 8.8%), and lumbar spine ( − 5.2%).^109^ Meta-analyses of caloric restriction trials have also confirmed that the degree of BMD loss is proportional to the magnitude of body mass reduction, especially at load-bearing sites like the femoral neck and total hip.^4,107,110^ For example, weight loss of ≥10% over 6–12 months has been associated with 1.0%–2.7% losses in hip BMD.^4,107^ These findings are further supported by RCTs in older adults demonstrating that dietary weight loss alone leads to a decline in BMD, while control groups maintain stable bone mass.^110–112^ Of note, increased daily physical activity, if not accompanied by sufficient body mass or targeted loading strategies, fails to restore net mechanical strain to pre-weight loss levels.^109^ This underscores the fact that mechanical input is not merely a function of movement frequency, but also of the magnitude and direction of the applied force.

In addition to gravitational loading, bone homeostasis is profoundly influenced by dynamic mechanical forces generated by skeletal muscle contractions. Muscle-bone interactions are crucial during movement and weight-bearing activities, where tensile and compressive forces are transmitted via tendons onto cortical and trabecular bone. These forces induce strain patterns that are essential for maintaining trabecular integrity, cortical thickness and periosteal apposition.^3,4^ During caloric restriction or sedentary behavior associated with weight loss, muscle mass and strength frequently decline. A randomized controlled trial in older obese adults demonstrated that participants in the diet-only group exhibited a 6.2% reduction in thigh muscle volume, whereas those in the exercise group saw a 2.7% increase. Most notably, changes in muscle volume were strongly correlated with changes in hip BMD (*r* = 0.55, *P* < 0.001) and served as an independent predictor of BMD loss after adjusting for age, sex, and weight change (β = 0.12, *P* = 0.03).^3^

Similar findings are supported by animal studies. In energy-restricted rodent models, preserved muscle mass through endurance exercise maintained total body BMC and femoral bone strength relative to sedentary counterparts, even under identical energy deficits.^4^ Additionally, long-term human cohort studies indicate that improvements in muscle strength (e.g., handgrip strength) are associated with attenuated bone loss in individuals undergoing weight reduction, whereas those with concurrent strength loss exhibit accelerated BMD decline.^113^ Therefore, muscle wasting not only reduces direct tensile and shear forces on bone, but also indirectly impairs the mechanosensory and osteogenic responsiveness of the skeletal system, particularly in older adults with reduced baseline mechanical competence.

Given that weight loss inherently reduces both body-mass–related and muscle-derived mechanical forces, exercise training represents the most effective and evidence-supported strategy to restore mechanical input to bone and counteract mechanical unloading. Numerous randomized controlled trials have demonstrated that structured exercise—especially resistance training and high-impact loading—can significantly attenuate BMD loss during weight loss interventions. For instance, in the LITOE trial involving older obese adults, participants undergoing resistance training in conjunction with a 10% weight loss program exhibited only a 0.7% reduction in hip BMD, compared to a 2.6% reduction in those assigned to aerobic exercise.^107^ Resistance training also elicited smaller increases in bone turnover markers, suggesting greater maintenance of bone remodeling balance. In a separate study involving obese women post-bariatric surgery, a 12-month descending stair training program (a high-impact, eccentric exercise) led to a 1.4% increase in femoral neck BMD, while control groups experienced marked losses. The stair training group also showed significant improvements in geometric bone strength indices such as cross-sectional moment of inertia and section modulus.^114^ These results demonstrate that only sufficiently intense, targeted, and multidirectional mechanical loading can effectively activate bone mechano-transduction pathways and stimulate new bone formation. Moreover, several studies highlight that exercise compliance, frequency, and training modality are critical moderators of skeletal outcomes. For example, in the RESOLVE study, individuals with high adherence to intensive exercise protocols experienced preserved or increased BMD, regardless of exercise type, whereas poor compliance negated the benefits.^115^ Conversely, interventions employing low-intensity, non-weight-bearing, or short-duration protocols generally fail to produce significant skeletal benefits.^116,117^

### BMAT accumulation

Although mechanical unloading is traditionally regarded as the primary drivers of bone loss, accumulating evidence suggests that BMD can also decrease in non–weight-bearing regions, and that BMD in these areas may exhibit a stronger association with weight loss than BMD in weight-bearing sites.^118–121^ This observation implies that additional factors likely contribute to the regulation of bone mass under weight loss conditions. Unlike white adipose tissue (WAT), particularly visceral fat, which typically decreases in mass and size during weight reduction, BMAT paradoxically increases.^122,123^ This distinction underscores the unique origin and physiological characteristics of BMAT compared to peripheral adipose depots. Furthermore, BMAT is increasingly recognized not as a passive space-filler within the marrow cavity, but as a metabolically active tissue capable of modulating bone homeostasis.

#### Energy deficiency alters bone–fat balance via BMSC fate switching

Prolonged caloric restriction or pathological energy deficiency, such as in anorexia nervosa or severe dietary restriction, leads to alterations in bone marrow stromal cell (BMSC) differentiation. Under these conditions, mesenchymal progenitors shift toward adipogenesis at the expense of osteogenesis, contributing to reduced bone formation and increased BMAT.^124,125^ In mouse models of separation-based anorexia (SBA), both the duration and severity of energy deficit (12%–24% body weight loss over 10 weeks) resulted in downregulation of Sirt1, a key regulator favoring osteoblastogenesis over adipogenesis. Decreased Sirt1 expression in BMSCs was accompanied by reduced osteoblast marker expression and enhanced adipogenic gene expression, despite no overt BMAT accumulation in the late stages.^124^ These findings are supported by CR mouse models, where 30% CR for 8 weeks resulted in a dramatic increase in BMAT and suppressed bone remodeling. Mechanistically, CR downregulated Wnt16 and canonical β-catenin signaling, impairing osteogenic commitment and promoting adipocyte differentiation.^125^ These shifts suggest that BMAT expansion under energy deficiency is not merely passive fat accumulation but an actively regulated adaptation via transcriptional remodeling of stem cell fate. To better elucidate the role of BMAT in this process, various genetic mouse models have been employed. In one study utilizing adiponectin-deficient (*Apn*⁻/⁻) mice, CR induced significant bone loss in wild-type (WT) animals but not in knockout (KO) counterparts, suggesting that adiponectin mediates CR-induced bone loss. Moreover, *Apn*⁻/⁻ mice exhibited resistance to BMAT accumulation following CR intervention.^126^ In our previous work, we further demonstrated that deletion of adipsin—another adipokine—similarly prevented CR-induced bone loss and BMAT expansion. BMSCs isolated from *Adipsin*⁻/⁻ mice displayed enhanced osteogenic differentiation capacity, accompanied by reduced adipogenic potential.^127^

#### BMAT expansion and bone loss are contextually linked but not always correlated

While BMAT is generally associated with decreased BMD and reduced skeletal quality,^5,6^ their relationship during weight loss is complex and may depend on the method of weight loss, site-specific responses, and metabolic state. In studies of bariatric surgery (RYGB and SG), BMAT responses diverged depending on surgical method and population characteristics. For instance, in postmenopausal, nondiabetic women undergoing RYGB, vertebral BMAT significantly decreased over 12 months, alongside vBMD reduction. Notably, no correlation was found between the magnitude of BMAT decline and BMD loss, suggesting independent regulatory mechanisms.^128^ Similarly, a study comparing RYGB and SG showed that MAT increased after SG but remained stable after RYGB, even though both procedures caused BMD reductions. Interestingly, in the SG group, patients who lost less visceral adipose tissue (VAT) exhibited greater increases in MAT, implying a compensatory or depot-specific lipid redistribution.^129^ Further complicating the picture, adolescent patients undergoing SG showed increased MAT in the axial skeleton(lumbar spine) but decreased MAT in peripheral sites, potentially reflecting age- and site-specific metabolic plasticity in marrow fat response.^130^

#### BMAT as an indicator and modulator of metabolic–skeletal crosstalk

Clinical MRI and spectroscopy studies reveal inverse associations between BMAT content and BMD across multiple populations, including healthy individuals, patients with osteoporosis, and those with obesity.^5^ BMAT’s role as a metabolic sensor is reinforced by observations that it expands during caloric deficiency and partially retracts following energy recovery, although this dynamic response is modulated by factors such as age, sex, diabetic status, and bone site.^5,6^ Moreover, BMAT lipid composition—particularly the saturation level of fatty acids—may influence osteoblast survival and bone remodeling, further supporting its mechanistic role in skeletal regulation.^5^ Weight loss strategies such as CR and MBS may differentially affect BMAT based on systemic metabolic changes, gut hormone shifts, and inflammation. While RYGB appears more likely to reduce BMAT in metabolically unhealthy individuals (e.g., diabetics), SG may increase BMAT, especially when VAT reduction is incomplete.^6^

### Hormonal and endocrine changes

#### Estrogen

Weight loss is an established risk factor for bone loss, particularly in women with low energy availability due to caloric restriction, excessive physical activity, or psychological stress. These conditions often lead to functional hypothalamic amenorrhea (FHA), characterized by hypoestrogenism. Low estrogen levels disrupt the balance between bone formation and resorption, thereby reducing BMD. This is especially concerning in adolescents, athletes, and patients with anorexia nervosa, where the failure to reach peak bone mass may have long-term skeletal consequences.^131–134^ Clinical data consistently indicate that hypoestrogenic women—regardless of baseline weight—demonstrate significantly lower BMD compared to eumenorrheic controls.^135–137^

Numerous studies have demonstrated the association between hypoestrogenism and site-specific BMD reduction. In adolescents with anorexia nervosa, lumbar spine BMD is significantly reduced and correlates with the duration of amenorrhea and level of physical activity.^135,137^ In a CT-based study of 24 women with FHA, spinal trabecular BMD was on average 20% lower than age-matched controls and inversely correlated with the duration of amenorrhea (*r* = −0.489, *P* = 0.02), while positively associated with circulating free testosterone and estradiol levels (*r* = 0.517, *P* = 0.02).^133^ Weight loss in premenopausal women also contributes to bone loss, particularly at the femoral neck, and this is exacerbated by low circulating estrone sulfate or estradiol levels.^138^ Post-bariatric surgery studies in adolescents revealed that bone loss at the spine and peripheral skeleton correlates with a decline in estrone and free androgen index (FAI).^139^ Prospective trials have further shown that weight loss combined with low estrogen levels accelerates hip BMD loss in both pre- and postmenopausal women.^140,141^ Further animal models provide mechanistic insight into how hypoestrogenism mediates bone loss during caloric restriction. In ovariectomized rats, energy restriction leads to a significant decline in serum estradiol, reduced intestinal calcium absorption (*r* = 0.68, *P* < 0.05), impaired calcium balance, and lower BMD. These effects can be mitigated by estradiol supplementation, highlighting its role in preserving bone metabolism under energetic stress.^142^ Moreover, lean rats exhibit more pronounced reductions in BMD under energy restriction than obese rats, likely due to greater estrogen depletion and lower 25(OH)D levels, suggesting that body composition influences estrogen’s protective capacity.^143,144^

Mechanically, estrogen influences bone homeostasis through a variety of tightly regulated pathways. In energy-restricted ovariectomized rats, a drop in estradiol levels leads to impaired calcium absorption, elevated urinary calcium excretion, and enhanced bone turnover, as demonstrated by increased markers of resorption and reduced bone mineral content.^142,143^ Lean animals are more susceptible than obese counterparts, indicating a heightened skeletal sensitivity to hypoestrogenism when body fat reserves are low.^144^ Clinical studies reinforce these observations: in FHA patients, bone density is negatively correlated with the duration of hypoestrogenism and positively with serum estradiol and testosterone levels.^133^ Beyond direct skeletal effects, estrogen modulates bone metabolism via interactions with metabolic hormones. In exercising women with FHA, hypoestrogenism is accompanied by reductions in IGF-1 and leptin and elevations in cortisol, creating a catabolic endocrine milieu that inhibits osteoblast activity and enhances osteoclastogenesis.^134,145^ Collectively, these findings suggest that estrogen plays a multifaceted role in regulating bone metabolism under energy-deficient states. It modulates the RANKL/OPG axis to suppress osteoclast activity, promotes osteoblast survival through PI3K/Akt and Wnt/β-catenin signaling, and maintains systemic calcium homeostasis via its effects on vitamin D metabolism and intestinal absorption. The loss of estrogen thus disrupts both the cellular and systemic regulation of bone remodeling, contributing significantly to weight loss-induced bone loss.

#### PTH

In addition to estrogen deficiency, PTH plays a pivotal role in the disruption of bone metabolism following bariatric surgery. Secondary hyperparathyroidism (SHPT) is a common and often persistent endocrine complication, particularly after malabsorptive procedures such as RYGB.^146^ Elevated PTH levels have been consistently associated with increased bone turnover and reduced BMD, especially at cortical-rich skeletal sites. Postoperative increases in PTH are multifactorial. Hypovitaminosis D remains highly prevalent in post-bariatric patients, with serum 25-hydroxyvitamin D [25(OH)D] levels below 20 ng/mL reported in up to 42% of individuals, despite routine supplementation.^147,148^ Impaired calcium absorption due to anatomical alterations stimulates PTH secretion to maintain serum calcium levels, contributing to SHPT. Elevated PTH, in turn, enhances the renal conversion of 25(OH)D to 1,25-dihydroxyvitamin D [1,25(OH)₂D], further depleting 25(OH)D reserves and promoting bone resorption.^146,147^ Importantly, SHPT is not a transient postoperative response but often persists over the long term. A retrospective study involving 350 bariatric patients found that SHPT was present in 72.9% at any point postoperatively, and 42.4% had persistent SHPT at 12 months despite adequate vitamin D3 supplementation.^149^ In a cohort study of 60 women approximately 7 years post-bariatric surgery, lower lumbar spine BMD was independently associated with decreased OPG/RANKL ratios and higher PTH levels.^150^ Few longitudinal cohort consistently showed that the prevalence of SHPT rose from 65.4% at year 2 to 83.7% by year 5 and 6 following RYGB, highlighting its chronic nature.^148,151^

Continuously elevated PTH levels have direct negative consequences for bone integrity.^152^ Multiple studies have reported inverse correlations between serum PTH and BMD at the femoral neck, lumbar spine, and total hip, regardless of weight loss or body composition.^153^ High-resolution imaging techniques reveal that cortical bone is particularly susceptible: PTH elevation is significantly associated with reductions in cortical thickness, cortical area, and density—particularly at weight-bearing sites such as the tibia—while trabecular bone is relatively spared in the early postoperative period.^16^ Beyond observational findings, experimental data support a mechanistic role of PTH in bone remodeling under energy deficit. In energy-restricted rodent models, intermittent low-dose PTH administration preserved both cancellous and cortical bone formation rates and prevented increases in bone marrow adiposity typically induced by caloric restriction.^154,155^ These findings highlight the dual action of PTH: while chronically elevated endogenous PTH is catabolic, pharmacologic PTH administered intermittently exerts anabolic effects on bone.

#### IGF-1

IGF-1 is a key anabolic regulator of bone metabolism. It promotes osteoblast proliferation, enhances collagen synthesis, and maintains bone remodeling balance. IGF-1 production is tightly linked to nutritional status, and its levels decline significantly in states of caloric restriction, rapid weight loss, or chronic undernutrition. In humans with AN, IGF-1 is one of the strongest hormonal predictors of low BMD, alongside estrogen and cortisol dysregulation. Cross-sectional and longitudinal studies show that IGF-1 correlates positively with osteocalcin and negatively with CTX, linking it closely with bone formation dynamics.^156^ During nutritional rehabilitation, especially in pre-menarcheal girls, increases in IGF-1 are independently associated with recovery of bone turnover markers, underscoring its essential role in pubertal bone accrual.^157^ Nutritional interventions, particularly protein intake, also modulate IGF-1 levels. In a 12-month randomized controlled trial among postmenopausal women undergoing caloric restriction, those assigned to a high-protein (HP) diet showed significantly higher serum IGFBP-3 and IGF-1 expression levels compared to a normal-protein (NP) group. The HP group also experienced reduced BMD loss at the hip, lumbar spine, and distal radius.^158^ These findings suggest that maintaining adequate protein intake during weight loss may mitigate skeletal deterioration by supporting IGF-1 signaling. Additionally, in a VSG mouse model, wild-type animals exhibited significant postoperative increases in insulin-like growth factor-binding protein 2 (IGFBP-2), which inhibits IGF-1 action. These mice experienced notable losses in areal and trabecular BMD, decreased bone volume fraction (BV/TV), and deterioration of cortical bone microarchitecture. In contrast, Igfbp2 knockout (*Igfbp2*⁻/⁻) mice were largely protected from these changes, demonstrating preserved trabecular BMD and attenuated cortical bone loss, despite similar weight and fat mass reductions.^159^ These findings implicate IGFBP-2–mediated suppression of IGF-1 as a mechanistic driver of post-surgical bone loss. In one of our ongoing studies, we observed that eight weeks of CR significantly reduced IGF-1 levels in both male and female mice. This decline may partially explain the more pronounced reduction in cortical bone compared to trabecular bone, given the critical role of IGF-1 in promoting periosteal apposition.^160,161^ Strategies to preserve IGF-1 availability—whether through dietary modification, suppression of IGFBP-2, or targeted pharmacologic approaches—represent promising avenues for preventing skeletal complications.

#### Leptin/ Adiponectin

Adipose tissue functions not only as an energy reservoir but also as an endocrine organ. Among the most studied adipokines are leptin, adiponectin, and resistin. During weight loss, the drastic reduction in fat mass leads to notable changes in circulating adipokine levels, which in turn influence bone. Leptin, primarily secreted by white adipose tissue, is a key mediator of appetite and energy expenditure via the hypothalamus.^162^ Clinical studies have revealed that leptin levels in serum significantly decrease with weight loss, concurrently with reductions in bone formation markers (e.g., BAP) and elevations in N-telopeptide.^163,164^ In addition, weight loss in young adults showed that bone microarchitecture, particularly in weight-bearing sites like the tibia, deteriorated after weight reduction. These changes were negatively associated with leptin levels, underscoring the potential mechanistic link between leptin deficiency and trabecular bone degradation.^165^ Animal studies using hypothalamic leptin gene therapy (via rAAV) demonstrated that leptin-induced weight loss did not cause dramatic difference in bone mass, density, microarchitecture, or biochemical bone markers.^166^ Nevertheless, leptin replacement at physiological doses during weight maintenance does not appear to reverse these changes, suggesting leptin’s bone-regulatory role may be secondary to overall metabolic state.^163^ Moreover, Adiponectin has anti-inflammatory and insulin-sensitizing effects and is also implicated in bone cell regulation. However, studies have reported elevated adiponectin levels in anorexia nervosa patients, which may cause reduced bone mass through inhibition of osteoblast activity and/or promotion of osteoclastogenesis.^162^ Similarly, increased adiponectin levels following bariatric surgery or dietary weight loss are related to decreased bone mineral density,^167^ although the exact mechanisms remain to be elucidated.

In addition to leptin and adiponectin, several other factors have been studied in the context of post-surgical bone metabolism, including periostin, sclerostin, and semaphorin 4D. Longitudinal observations indicate that these factors exhibit delayed increases after weight loss, of modest magnitude. Their temporal patterns suggest these may play a role in skeletal adaptation but are unlikely to be primary drivers of bone loss following weight loss.^167^

#### Gastrointestinal hormones

Bariatric surgery alters the gastrointestinal environment, including hormonal responses and microbial composition. These changes, while beneficial for weight loss and metabolic improvement, have significant implications for bone health. Recent studies indicated that surgical interventions for weight loss markedly increase the secretion of gut-derived hormones including GLP-1, peptide YY (PYY), and decrease ghrelin levels. These hormones influence bone remodeling through direct and indirect mechanisms. GLP-1 is one of the most markedly elevated incretin hormones following bariatric surgery, secreted primarily by L-cells in the distal ileum and colon in response to nutrient stimulation. After surgery, GLP-1 secretion increases substantially, and may indirectly influence osteoblast and osteoclast activity via its receptor.^140,168–171^ Animal studies have shown that GLP-1 receptor knockout mice exhibit decreased bone mass and reduced skeletal quality, suggesting that GLP-1 promotes bone formation and suppresses bone resorption.^171^ As noted, GLP1RAs can negatively impact the skeleton. A recent study comparing VSG with GLP-1RA treatment (semaglutide) in obese mice revealed important differences in skeletal outcomes. While both interventions produced comparable weight loss and improved glucose metabolism, VSG led to significant bone loss, whereas 6 weeks of semaglutide treatment did not result in bone deterioration, despite similar reductions in body weight.^75^ However, prolonged treatment may still lead to reductions in BMD at sites such as the hip and spine,^172^ indicating that hormonal regulation may be influenced by factors including weight status and nutrient availability. PYY, another satiety hormone, is primarily secreted by L-cells in the distal ileum and colon. Postprandial PYY secretion is significantly increased after bariatric procedures, where it acts on the hypothalamus to suppress appetite and delay gastric emptying.^140,168,169^ Notably, elevated PYY levels are associated with increased bone turnover markers, including CTX and P1NP,^140^ suggesting its role in promoting a high bone turnover state postoperatively. However, the underlying mechanisms remain unclear. Ghrelin, predominantly secreted by P/D1 and x/a cells in the gastric fundus, is currently the only known peripheral hormone that stimulates appetite. Its levels decrease significantly following SG and RYGB,^170^ and this reduction correlates with declines in lumbar spine BMD.^170^ Ghrelin has been shown to stimulate osteoblast proliferation and mineralization, and its reduction following bypass surgery may therefore impair bone formation, contributing to postoperative bone loss. Interestingly, in conditions of energy deficiency such as AN, ghrelin levels are increased. This phenomenon, observed in both animal models and patients, likely reflects hormonal resistance or impaired downstream signaling, contributing to persistent bone loss and depleted energy reserves.^173^ These findings imply that ghrelin’s skeletal effects are modulated by the metabolic state and may involve desensitization or signal reprogramming. Moreover, Glucose-dependent insulinotropic polypeptide (GIP) levels also rise postoperatively after gastric bypass. Animal studies suggest that GIP enhances bone formation and slows resorption,^171^ although evidence in humans remains limited. In addition to hormonal adaptations, bariatric surgery induces notable shifts in the gut microbiota, which may mediate skeletal changes through multiple pathways. Studies utilizing 16S rRNA sequencing revealed that in Laparoscopic Sleeve Gastrectomy (LSG) patients the changes in microbial β-diversity were positively correlated with increased P1NP and negatively correlated with femoral neck BMD.^174^ Moreover, decreased fecal butyrate—a short-chain fatty acid (SCFA) produced by gut bacteria—was associated with lower IGF-1 levels and higher CTX concentrations, suggesting that microbial metabolites may influence bone turnover via IGF-1-mediated pathways.^174^ In contrast, clostridium butyricum supplementation in rats following RYGB mitigated bone loss by promoting osteoblast autophagy and counteracting microbiota-induced skeletal disruption,^175^ highlighting its therapeutic potential. Furthermore, dietary intervention studies have shown that reductions in trimethylamine N-oxide, another gut microbiota-associated metabolite, are linked to significant loss in whole-body and spine BMD during weight loss, independent of diabetes status or diet composition.^176^

#### Other factors

Fibroblast growth factors FGF21 and FGF15/19 play key roles in metabolic regulation and have emerging relevance to bone health during weight loss. FGF21 has been demonstrated to promote weight loss and enhance glucose and lipid metabolism. In obese rhesus monkeys. 12 weeks of FGF21 treatment resulted in significant reductions in body weight and improved insulin sensitivity. However, it also induced a two-fold increase in CTX-1, a marker of bone resorption, without producing measurable changes in BMD. This suggests that FGF21 may promote bone resorption even in the absence of short-term BMD loss.^177^ In contrast, FGF15 appears to have a protective role following bariatric surgery. In intestinal FGF15 knockout mice, VSG caused greater loss of lean mass and BMD compared to controls. These mice also showed impaired glucose tolerance and elevated bile acid levels post-surgery, indicating that FGF15 helps preserve bone and metabolic stability after VSG.^175^

### Vitamin D & Ca^2+^

During weight loss, particularly following metabolic bypass surgeries patients often are vitamin D deficient with secondary lowering of serum calcium; these are closely related to bone loss. On the one hand, numerous studies have shown that individuals with obesity are inherently prone to low serum levels of 25OH vitamin D. This is likely due to the sequestration of fat-soluble vitamin D within adipose tissue, reduced sun exposure, and abnormal hepatic activation of 25OH vitamin D. After weight loss, although the reduction in adipose tissue may release some “trapped” vitamin D, vitamin D levels often further decline due to impaired intestinal absorption, particularly post-surgery.^147,178,179^ In a study of 136 patients who underwent malabsorptive procedures, 34% had severe vitamin D deficiency, and 42% had mild to moderate deficiency, which was positively associated with postoperative BMD decline.^147^ On the other hand, impaired calcium absorption is another key metabolic disruption after weight loss. Surgical procedures, especially those bypassing the jejunum or ileum, directly reduce the absorptive surface area for calcium. Even with adequate dietary calcium intake, serum calcium levels may remain low or low-normal. To maintain calcium homeostasis, PTH secretion increases, initiating bone resorption and further leading to bone loss.^143,180^

Multiple clinical trials and basic studies have evaluated the effectiveness of vitamin D and calcium supplementation during weight loss. Overall, supplementation can moderately improve bone metabolism markers, suppress SHPT, and slow bone loss, but its efficacy is limited by dosage, timing, individual differences, and surgical type, and cannot completely prevent bone loss. Calcium supplementation is a key strategy to control bone loss during weight loss. Studies show that 1.2–1.7 g/day calcium intake can significantly reduce BMD loss in the hip and spine and suppress PTH elevation.^86,181,182^ In one low-calorie diet study involving 62 obese women, those receiving calcium supplementation had significantly less spinal BMC loss than controls.^183^ Another study found that among overweight postmenopausal women, bone loss persisted with 1 g/day calcium but was mitigated when the dose was increased to 1.7 g/day.^181^ Calcium also affects bone turnover markers. Supplementation reduces resorption markers such as CTX, DPD, and OPG, helping preserve trabecular bone structure.^155,182^ Especially during the high turnover phase post-surgery, calcium supplementation helps stabilize bone metabolism and curb excessive remodeling.

Adolescents, who are undergoing peak bone mass accumulation, have active bone metabolism and may be more susceptible to weight-loss–related changes. One study showed that 12 months after SG, adolescents had significant declines in hip and femoral neck aBMD Z-scores, trabecular number, widened trabecular spacing, and reduced cortical thickness, despite stable serum 25(OH)D levels.^86^ Postmenopausal women, already at increased risk of bone loss due to estrogen deficiency, experience exacerbated metabolic disturbances from weight loss. One study found that with the same calcium intake (1 g/day), overweight postmenopausal women (BMI 27–28) experienced greater BMD loss during calorie restriction compared to obese women (BMI > 30).^181^ Besides, Individual hormonal environments also affect vitamin D and calcium metabolism during weight loss. A comparative study of obese and lean rats found that lean rats showed greater drops in estrogen and vitamin D and higher urinary calcium excretion after weight loss, suggesting increased sensitivity to calcium disturbances.^143^ Postoperative changes in hormones such as PYY, leptin, and insulin also regulate bone remodeling and mineral absorption.^143,179^

Furthermore, different bariatric surgeries also affect vitamin D and calcium metabolism to varying degrees, depending on changes in gut anatomy and absorption function. Malabsorptive procedures (e.g., RYGB, BPD) significantly reduce absorption areas by bypassing the jejunum or ileum, leading to more severe nutrient deficiencies and bone loss.^147,179,180,184^ Clinical follow-up also shows that despite standardized supplementation, RYGB and BPD patients have more pronounced and persistent bone loss compared to other procedures.^182,185^ A clinical trial on OAGB patients found that despite maintaining 25(OH)D > 50 nmol/L, high bone turnover led to whole-body and hip BMD declines within 12 months.^186^ In contrast, restrictive procedures like SG have less impact on the small intestine but may still disrupt calcium metabolism through reduced gastric acid and increased PTH.^86,180^ This suggests that supplementation alone may not reverse the profound metabolic changes caused by malabsorptive surgery, necessitating tailored interventions.

Variations in dietary patterns—including energy restriction, protein sources, calcium intake, and micronutrient supplementation—can also result in significantly different effects on bone metabolism.^187^ High-fiber diets may assist in weight control but have been shown to accelerate lumbar spine bone density loss in postmenopausal women, and bone mass often fails to recover even after weight regain.^9^ The effects of high-protein diets remain controversial.^188^; Beyond macronutrients, the interaction between caloric restriction and micronutrient deficiencies is another key mechanism driving bone loss during weight reduction. Caloric restriction without adequate calcium intake leads to enhanced bone resorption, though supplementation with dairy products can mitigate this trend.^189,190^ Meanwhile, long-term high-protein intake may increase urinary calcium excretion but may also raise IGF-I and protect the skeleton from weight loss-induced bone loss.

## Conclusion and Future Perspectives

Weight loss, while beneficial for reducing cardiometabolic risk, is consistently associated with adverse effects on skeletal health. Across different modalities—including caloric restriction, exercise-based interventions, pharmacological agents, and bariatric surgery—weight reduction has been shown to result in bone loss, reduced BMD, and compromised bone quality. These skeletal consequences are particularly concerning given the global rise in obesity, increased use of weight loss interventions, and the aging of the population. Mechanistically, bone loss during weight reduction can be attributed to a combination of factors. Nutritional deficiencies, especially in calcium and vitamin D, are common after bariatric procedures and contribute to secondary hyperparathyroidism, enhanced bone turnover, and accelerated bone resorption. Hormonal alterations, including reduced gonadal, thyroidal, and somatotropic activity, further impair bone formation and promote catabolism of lean tissues, including bone. Additionally, weight loss leads to reduced mechanical loading on the skeleton, which disrupts bone remodeling via mechanosensitive pathways. The expansion of BMAT under conditions such as caloric restriction may also play a role by altering the bone marrow niche and inhibiting osteoblastogenesis through adipocyte-derived cytokines.

Despite the implementation of calcium and vitamin D supplementation during weight loss, these measures are often insufficient to fully counteract skeletal deterioration. Taken together, weight loss induced bone loss represents a clinical challenge that is likely to be more prevalent as anti-obesity treatments become more widely available. This highlights the need for integrated strategies that preserve bone mass while achieving overall metabolic health.
